# Identification of genomic determinants contributing to cytokine release in immunotherapies and human diseases

**DOI:** 10.1186/s12967-022-03531-3

**Published:** 2022-07-28

**Authors:** Lipei Shao, Alejandra Pelayo, Rongye Shi, Jinxia Ma, Hui Liu, Yihua Cai, Michaela Prochazkova, Robert P. Somerville, Sandhya R. Panch, Nirali N. Shah, David F. Stroncek, Ping Jin

**Affiliations:** 1 Department of Transfusion Medicine, Center for Cellular Engineering, NIH Clinical Center, Bethesda, MD 20892 USA; 2 Pediatric Oncology Branch, Center for Cancer Research, NIH NCI, Bethesda, MD 20892 USA

**Keywords:** CAR T-cell therapy, Cytokine release syndrome, PFKFB4, Glycolysis

## Abstract

**Background:**

Cytokine release syndrome (CRS) is a strong immune system response that can occur as a result of the reaction of a cellular immunotherapy with malignant cells. While the frequency and management of CRS in CAR T-cell therapy has been well documented, there is emerging interest in pre-emptive treatment to reduce CRS severity and improve overall outcomes. Accordingly, identification of genomic determinants that contribute to cytokine release may lead to the development of targeted therapies to prevent or abrogate the severity of CRS.

**Methods:**

Forty three clinical CD22 CAR T-cell products were collected for RNA extraction. 100 ng of mRNA was used for Nanostring assay analysis which is based on the nCounter platform. Several public datasets were used for validation purposes.

**Results:**

We found the expression of the PFKFB4 gene and glycolytic pathway activity were upregulated in CD22 CAR T-cells given to patients who developed CRS compared to those who did not experience CRS. Moreover, these results were further validated in cohorts with COVID-19, influenza infections and autoimmune diseases, and in tumor tissues. The findings were similar, except that glycolytic pathway activity was not increased in patients with influenza infections and systemic lupus erythematosus (SLE).

**Conclusion:**

Our data strongly suggests that PFKFB4 acts as a driving factor in mediating cytokine release in vivo by regulating glycolytic activity. Our results suggest that it would beneficial to develop drugs targeting PFKFB4 and the glycolytic pathway for the treatment of CRS.

**Supplementary Information:**

The online version contains supplementary material available at 10.1186/s12967-022-03531-3.

## Introduction

Cytokine release is a systemic inflammatory response that occurs when immune cells are activated by foreign substances and release large amounts of cytokines into the body such as: interleukins, interferons, tumor necrosis factors and growth factors [1, 2]. All of these cytokines are small proteins which play a vital role in host defense when regulated normally. However, when the immune response is dysregulated, high levels of cytokines may be produced which causes increased inflammation throughout the body and which results a condition called cytokine release syndrome (CRS). CRS can present with a variety of symptoms ranging from mild to severe and life-threatening manifestations [3, 4]. Mild symptoms of CRS include cough, fever, headache, fatigue, rash, and myalgia [1]. While severe cases are characterized by hypotension and high fever which may progress to an uncontrolled systemic inflammatory response resulting in vascular leakage, circulatory shock, and multi-organ system failure [1]. Thus, effective treatment is required when patients develop severe CRS.

To date, CRS has been observed in infectious diseases (pandemics of influenza [5, 6], SARS-CoV and COVID-19 [7, 8]), certain acquired or inherited autoimmune diseases [9, 10], and following chimeric antigen receptor T-cell (CAR T-cell) therapy [11, 12]. Patients with severe CRS are treated with therapies designed to block specific cytokines, as well as more general immunosuppressive drugs. The anti-cytokine drugs, tocilizumab and siltuximab (anti-IL-6 receptor antibodies) have been widely used to minimize rates of life-threatening CRS in patients receiving CAR T-cell therapy and in patients with severe COVID-19 [13–15]. Corticosteroids such as methylprednisolone or dexamethasone, are also used to help mitigate inflammatory and immune responses by providing broad immunosuppression in individuals with autoimmune diseases and COVID-19 [16–18]. However, while the usage of tocilizumab may decrease CRS severity, it may increase the risk of neurotoxicity, another common toxicity during CAR T-cell therapy [19, 20]. In addition, not all patients with severe CRS respond well to tocilizumab or corticosteroid treatments [21, 22], thus pre-emptive strategies are being tested [23, 24]. An early clinical trial showed a 69% response rate to tocilizumab in patients with severe or life-threatening CRS [22]. As for corticosteroids, there is still conflicting data as to whether its use compromises CAR T-cell potency [25, 26]. Improved CRS treatment and prevention requires a greater understanding of molecular and cellular determinants contributing to cytokine release. The current understanding of factors that trigger and drive cytokine release remains incomplete.

In recent years, the Nanostring nCounter gene expression platform has emerged and developed quite quickly [27–29]. It is a high-fidelity, simple protocol that allows for the detection of 800 mRNA molecules of interest at one time using specific probes. This method has proven to be simpler and more effective compared to real-time qPCR, and time-saving and easier to analyze compared to RNA-seq [30, 31]. Here, in order to explore candidate targets for CRS treatment, we systematically explored the genomic factors found in CAR T-cell clinical products using the Nanostring nCounter platform. In this study, we analyzed 43 pre-infusion CD22 CAR T-cell products and their corresponding clinical CRS grade. After analyzing gene expression using the nSolver software, we compared the differentially expressed genes and pathways among CAR T-cell products associated with different CRS grades. We found that the PFKFB4 gene and its regulated glycolytic pathway activity were gradually upregulated among CAR T-cell products grouped from mild to severe CRS. Moreover, using public datasets we validated our results in several other human diseases where CRS can occur including: COVID-19, influenza, autoimmune diseases and human tumors. These analyses suggest that the PFKFB4 gene may act as a driving factor in triggering the cytokine release process. Drugs targeting PFKFB4 and glycolytic pathway or a combination strategy might be beneficial for the clinical management of cytokine release syndrome.

## Materials and methods

### CAR T-cell products

CD22 CAR T-cell products were obtained from excess products manufactured for patients enrolled on a phase I trial of CD22 CAR T-cells in B-cell malignancies. (Clinicaltrials.gov NCT02315612). The clinical trial was approved by the National Cancer Institute Institutional Review Board. Products that were analyzed were from patients who consented to additional genomic testing and were enrolled on a companion study for study of biologic correlatives (Clinicaltrials.gov NCT01109394). CRS was graded using ASTCT consensus grading [32].

### mRNA extraction

Total RNA was isolated from 43 cryopreserved pre-infusion CD22 CAR T-cell products [33] using miRNeasy Mini Kit (Qiagen). Quantity and Quality were measured by Nanodrop 8000 (Thermo Fisher Scientific) and 2100 Bioanalyzer (Agilent).

### Gene expression profiling by Nanostring

Hundred ng of total RNA was hybridized in solution with the nCounter CAR-T Characterization panel (human codeset) at 65 ℃ for 17 h. The hybridized samples were loaded into the nCounter CAR-T cartridge (NanoString Technologies), which was then sealed and placed in the instrument for processing and analysis. RCC files containing raw counts for 780 genes were generated and loaded into nSolver Analysis Software 4.0 for normalization by housekeeping genes and positive controls. The normalized data were imported to Partek Genomics Suite 7.0 to remove any batch effects. Downstream analysis was performed in a Rstudio environment with custom code.

### Calculation of differentially expressed genes

The limma R package was used to generate p-values and fold change (FC) for each gene between samples with different CRS grade. Wald’s Test was used to calculate the *p* value or significance that a gene is differentially expressed. Genes with a *p*-value ≤ 0.01 and a │log_2_ (FC)│ ≥ log_2_(1.5) were identified as differentially expressed genes (Additional file 4: Table S1).

### Gene set analysis

Gene set variant analysis (GSVA) was used to look at enrichment scores for custom pathways in order to check pathway activity based on GSVA R package. *p*-value under 0.05 represents statistical significance.

### Public datasets

Publicly available gene-expression profiles were used to validate findings in our studies were downloaded from GEO and TCGA datasets. Among them, cells were collected from whole blood (GSE196822, GSE111368 and GSE72326), peripheral blood mononuclear cells (GSE152418, GSE179627 and GSE114588), and monocytes (GSE147608). PFKFB4 gene expression data and association with immune cells in pan-cancer downloaded from TIMER website.

### Statistical analysis

All statistical analysis was performed with GraphPad Prism software and related R package. A p-value less than 0.05 was considered significant.

## Results

### PFKFB4 gene expression is gradually upregulated among CAR T-cells grouped according to recipient cytokine release syndrome grade

**Fig. 1:**
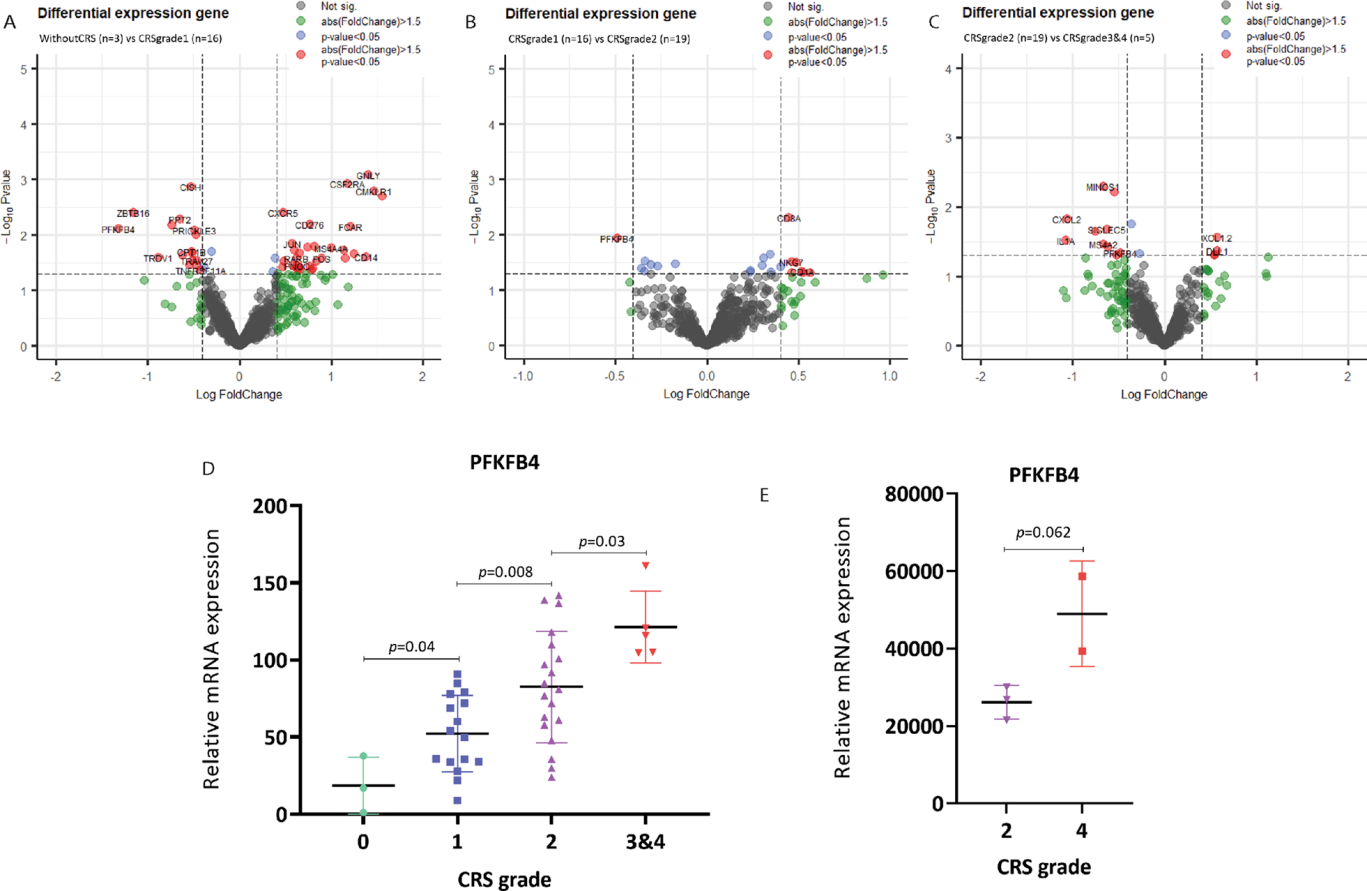
PFKFB4 gene is gradually up-regulated with the development of cytokine release syndrome in CAR T-cell therapy. **A**–**C** Volcano plot of CRS grade-related differentially expressed genes between the different groups. Fold Change > 1.5 and *p*-value < 0.05 were set as screening criteria. Genes that have both a significant *p*-value (lower than 0.05) and a fold change (higher than 1.5) are represented as red dots. Genes that either have a significant *p*-value (lower than 0.05) or a fold change (higher than 1.5) are represented as blue and green dots. Gray dots mean genes neither have a significant *p*-value nor fold change. PFKFB4 gene expression in CD22 CAR T-cell (**D**) and CD19 CAR T-cell products (**E**) based on different CRS grade group

In order to identify gene expression signatures that drive the development of CRS, we collected 43 pre-infusion CD22 CAR T-cell products and analyzed gene expression using the Nanostring nCounter gene profiling system. First, we divided the CAR T-cell samples into four groups based on maximum clinical CRS grade in the associated recipient: Without CRS (n = 3), CRS grade 1 (n = 16), CRS grade 2 (n = 19), CRS grade 3 and 4 (n = 5). Then, we compared differentially expressed genes between 3 combinations of two groups (without CRS vs CRS grade 1, CRS grade 1 vs CRS grade 2, CRS grade 2 vs CRS grade 3 and 4) in order to find genes whose expression gradually changed as CRS great increased. As the volcano plot shows in Fig. 1A–C, we identified some differentially expressed genes based on different comparisons (Additional file 4: Table S1). Interestingly, we found that the PFKFB4 gene was gradually up-regulated as CRS became more severe (Fig. 1D).

Based on this finding, we sought to explore other CAR T-cell trials. Based on data generated from a CD19 CAR T-cell trial, we found similar results [34]. Based on this study’s supplementary data, we extracted the PFKFB4 gene expression and CRS grade information. As shown in Fig. 1E, PFKFB4 gene expression was up-regulated in patients receiving CD19 CAR T-cells with grade 4 CRS (*p* = 0.062). Our results indicate that PFKFB4 gene may act as an essential factor in triggering the development of cytokine release syndrome.

### PFKFB4 triggers cytokine release through the regulation of glycolytic activity

**Fig. 2:**
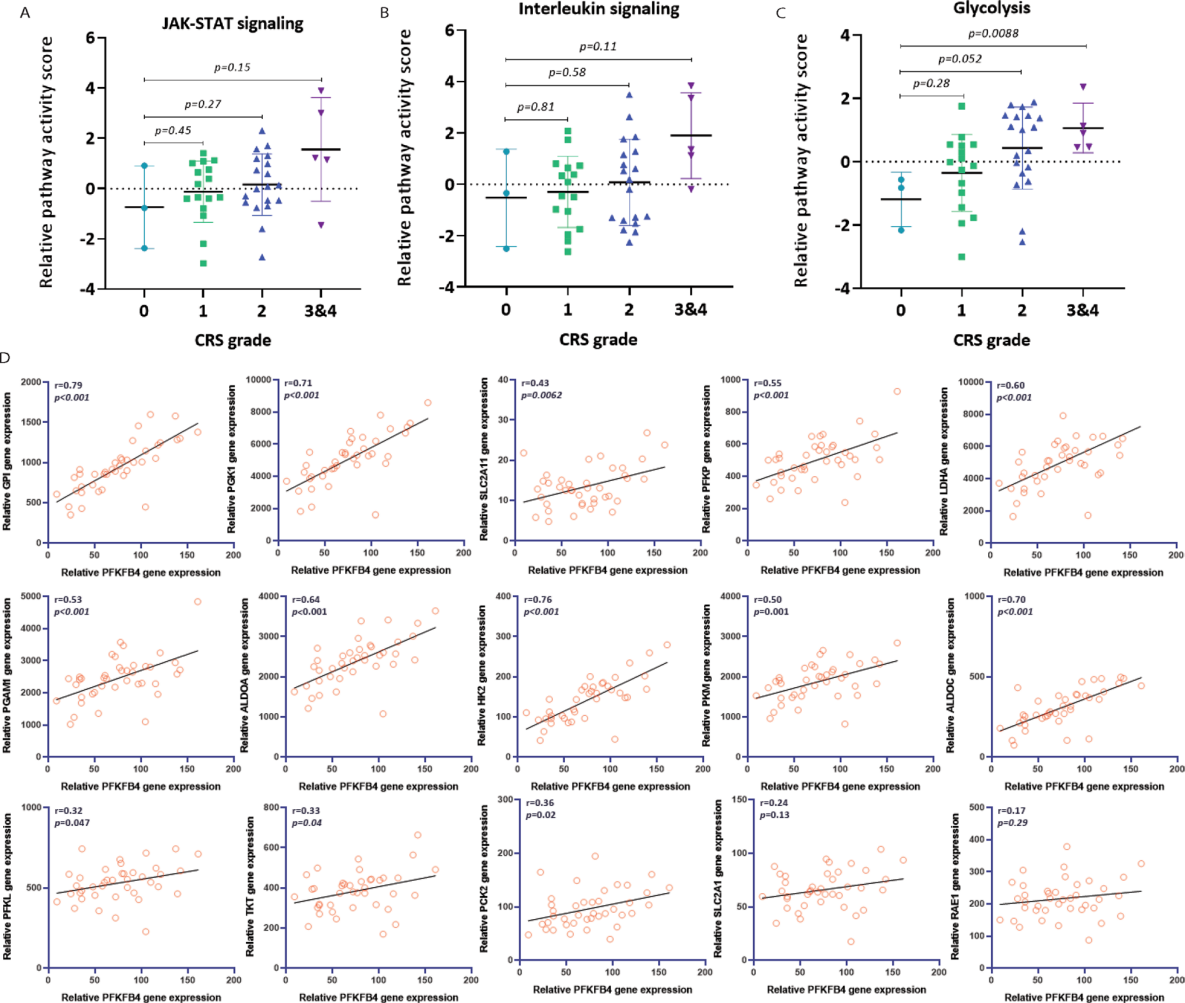
Glycolytic pathway activity is enhanced in groups with higher CRS grades. **A**–**C** Relative signaling pathway activity score was calculated based on GSVA analysis. JAK-STAT, Interleukin, and glycolysis signaling pathways showed higher activity in the high CRS grade group. The x-axis represents different CRS grades. **D** Correlation between PFKFB4 gene expression and genes involved in glycolysis signaling pathway. Correlation coefficient and *p*-values are listed at the up-left corner. The x-axis represents relative PFKFB4 gene expression. The y-axis shows the expression of genes that are involved in the glycolysis pathway

It has been reported that interleukin and JAK-STAT signaling pathways play a key role in the cytokine release process and blocking/inhibiting these pathways can significantly reduce the severity of CRS induced by CAR T-cells [35]. Consequently, we explored additional key signaling pathways in order to facilitate the development new targeted therapies. As described in Fig. 1, four groups were divided to compare each signaling pathway activity. We selected 18 related signaling pathways, including JAK-STAT and interleukin signaling. First, we recognized the trend that JAK-STAT and interleukin signaling pathway activity was gradually enhanced with higher statistical power during the development of more severe CRS (Fig. 2A and B, Additional file 1: Fig. S1). In addition, a similar and more obvious trend was observed in glycolytic activity (Fig. 2C). We found that the expression of most genes involved in glycolytic signaling pathway were positively correlated with PFKFB4 gene expression (Fig. 2D) including the important regulators of glycolytic process GPI, PGK1, and PKM. Given these results, we speculated that PFKFB4 is a driving factor that plays a vital role in the development of cytokine release possibly through regulation of glycolytic pathway activity.

### PFKFB4 expression and glycolytic pathway activity is enhanced in other diseases associated with cytokine release

**Fig. 3:**
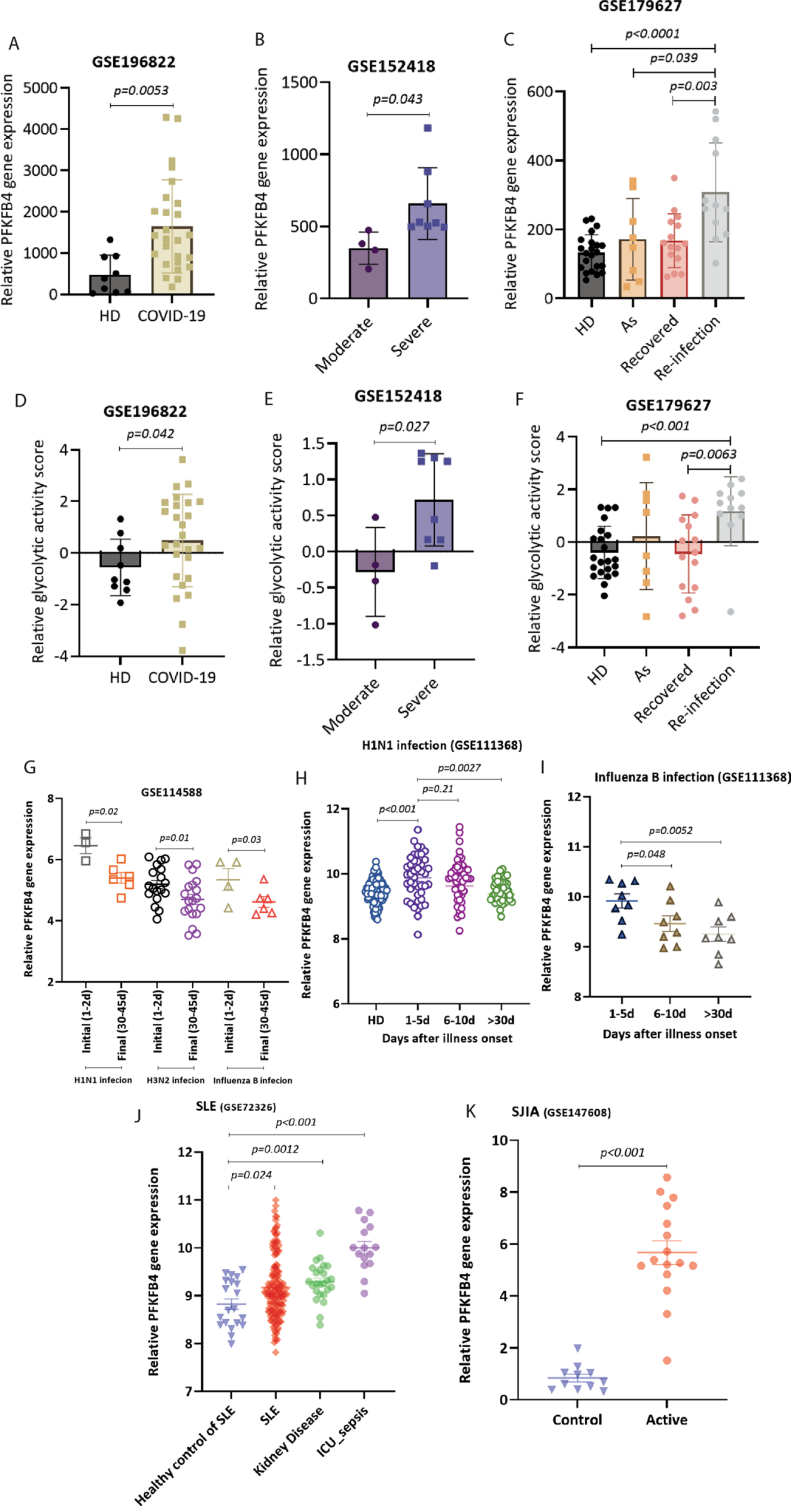
PFKFB4 expression and glycolytic pathway activity is also enhanced in some human diseases associated with cytokine release syndrome. PFKFB4 gene expression profile in COVID-19 patients vs healthy donor (**A**), COVID-19 patients with severe symptoms vs moderate symptoms (**B**), healthy donors vs asymptomatic vs recovered vs re-infected COVID-19 patients (**C**). Glycolytic activity score in COVID-19 patients vs healthy donors (**D**), COVID-19 patients with severe symptoms vs moderate symptoms (**E**), healthy donors vs asymptomatic vs recovered vs re-infection COVID-19 patients (**F**). PFKFB4 gene expression in people infected with influenza based on early and late stage infection (**G**–**I**). PFKFB4 gene expression in different stages of SLE (**J**) and SJIA (**K**). *HD* healthy donor, *As* asymptomatic, *SLE* systemic lupus erythematosus, *SJIA* systemic juvenile idiopathic arthritis

Currently, a considerable number of patients with COVID-19 have developed cytokine release syndrome and the resulting CRS severity positively correlated with the pathogenesis and severity of COVID-19. To validate our findings in COVID-19 cohort, we analyzed three public datasets from the GEO platform. In general, PFKFB4 gene expression was significantly up-regulated in COVID-19 patients compared to healthy donors (Fig. 3A). Moreover, its expression was higher in patients with severe syndrome compared to patients with moderate syndrome (Fig. 3B). In addition, we noted that PFKFB4 gene expression in asymptomatic people infected with SARS-CoV-2 and in COVID-19 convalescent people were stable and at the same level as in healthy subjects. While, PFKFB4 expression increased following re-infection by SARS-CoV-2 (Fig. 3C). Concerning glycolytic pathway activity, the results showed that pathway activity also increased during SARS-CoV-2 infection and COVID-19 development (Fig. 3D–F).

Since people with seasonal influenza infections and autoimmune diseases can also develop CRS, we explored PFKFB4 expression in these diseases. As shown in Fig. 3G–I, we found that PFKFB4 expression was up-regulated in influenza-infected (H1N1, H3N2, Influenza B) individuals in the early stages of infection, and decreased in the recovery stage. Among autoimmune diseases, we selected systemic lupus erythematosus (SLE) and systemic juvenile idiopathic arthritis (SJIA) for further validation. We found that patients with severe or active disease showed higher PFKFB4 gene expression (Fig. 3J and K). However, glycolytic pathway activity showed mixed results. Its activity was lower in early stage of influenza-infected patients and SLE patients with severe status (Additional file 2: Fig. S2A–D), while it was higher in SJIA patients with active disease status (Additional file 2: Fig. S2E).

These results provide further validation of our findings that PFKFB4 gene and glycolytic pathway play a key role in cytokine release.

### Up-regulated PFKFB4 gene and glycolytic activity during tumor development

**Fig. 4:**
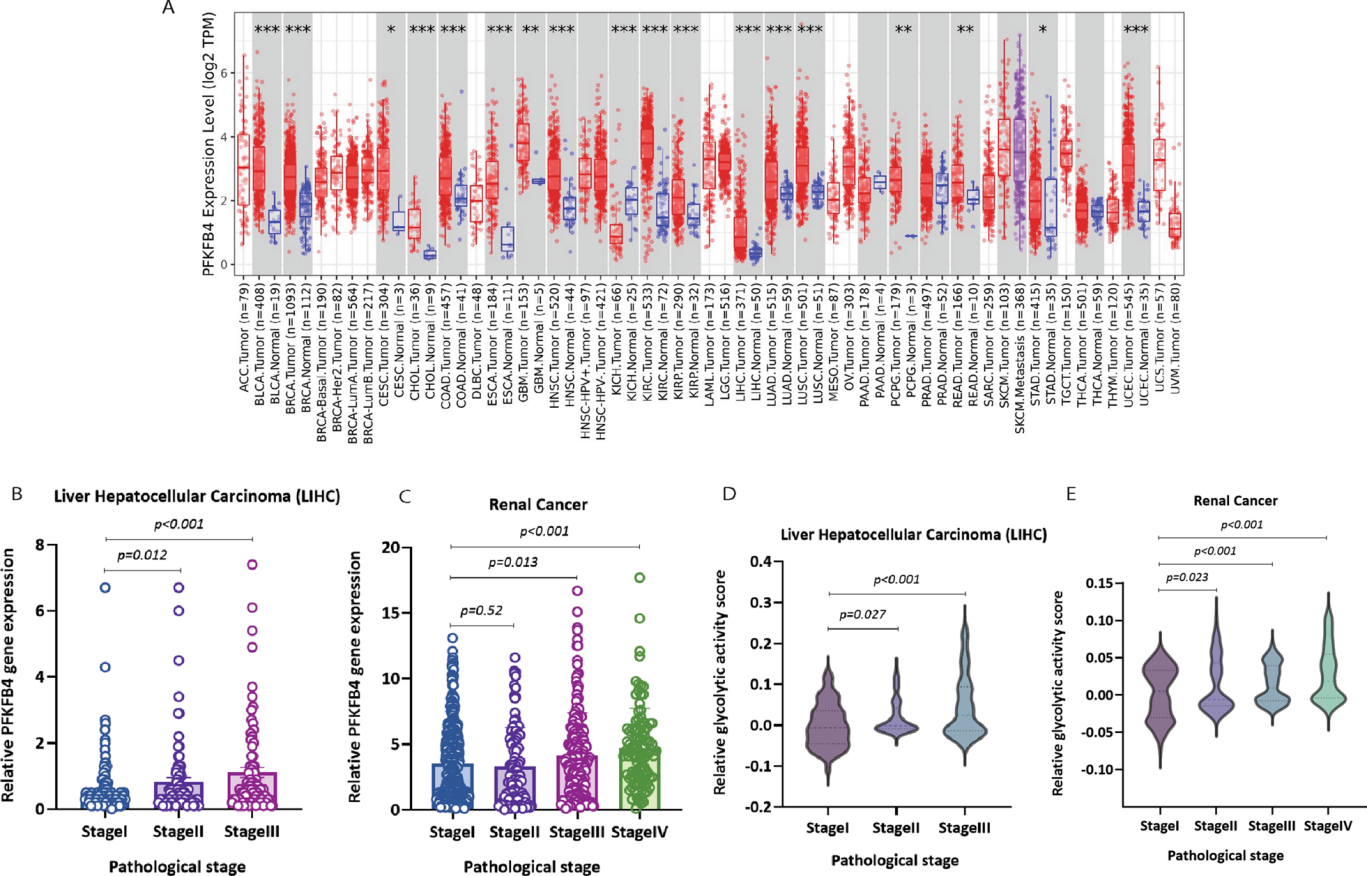
The relationship between the level of PFKFB4 expression and clinicopathological stages in cancer. **A** Overview of PFKFB4 gene expression in all cancers. Red represents tumor tissue, blue represent adjacent normal tissue. **B**, **C** Upregulation of the PFKFB4 gene in higher pathological stage of liver hepatocellular carcinoma and renal cancer. **D**, **E** Enhanced activity of glycolysis in different pathological stage in liver hepatocellular carcinoma and renal cancer. The x-axis represents different pathological stages of the tumors

It has been widely accepted that various cytokines are released into the tissue microenvironment during the tumorigenesis and tumor development process. These cytokines may inhibit tumor development but alternatively may contribute to the chronic inflammation that supports tumor growth and has been linked to poor clinical outcomes [36]. In order to investigate whether the PFKFB4 gene is also involved in the cytokine-mediated inflammatory microenvironment and subsequent metastasis process in tumor tissues, we evaluated the correlation of PFKFB4 expression and tumor pathological stages in several types of cancer. First, we used the Tumor Immune Estimation Resource (TIMER2.0) database to explore the expression of PFKFB4 in several cancers. We found its expression was significantly up-regulated in 18 cancer types (i.e., breast invasive carcinoma, liver hepatocellular carcinoma, colon adenocarcinoma, kidney renal clear cell carcinoma, kidney renal papillary cell carcinoma) when compared with corresponding normal tissues (Fig. 4A). Furthermore, we analyzed the correlations between PFKFB4 expression and pathological stages in several tumor types. We selected liver hepatocellular carcinoma and renal cancer, which includes kidney chromophobe, kidney renal clear cell carcinoma, and kidney renal papillary cell carcinoma. We found PFKFB4 gene was significantly up-regulated in patients with higher pathological stage (Fig. 4B and C) and shorter survival time (Additional file 3: Fig. S3A and B). Furthermore, the activity of the glycolytic pathway was also enhanced in patients with late pathological stage (Fig. 4D and E). Moreover, we found PFKFB4 gene expression showed a strong correlation with genes involved in glycolytic pathway in all cancers studied (Additional file 3: Fig. S3C). These data indicate that the PFKFB4 gene regulates glycolytic activity and can promote tumor metastatic process possibly through the induction of a pro-inflammatory microenvironment in tumor tissue by mediating cytokine release.

### PFKFB4 induces a pro-inflammatory microenvironment in cancer

**Fig. 5:**
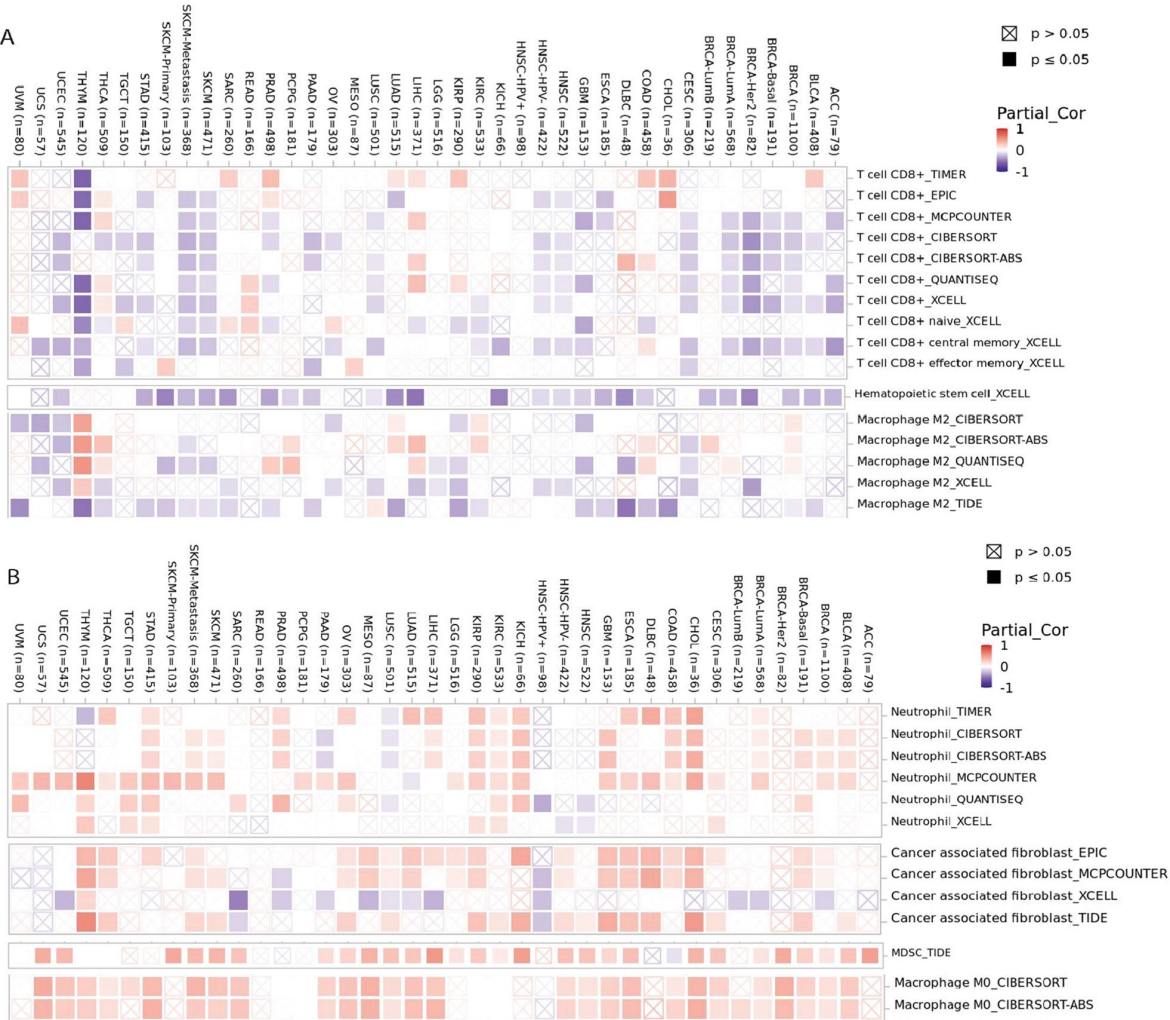
Association between PFKFB4 expression and tumor immunity several types of cancer. **A** Correlations of PFKFB4 gene expression with CD8 + T cells, hematological stem cells, and M2 macrophages. **B** Correlations of PFKFB4 gene expression with neutrophil, cancer associated fibroblast, myeloid-derived suppressor cells, and M0 macrophages. Purple and red colors represent negative and positive correlations

To further validate PFKFB4 expression and its role in pro-inflammatory microenvironment in tumors, we analyzed PFKFB4 expression in assorted immune cell infiltration in several algorithms including CIBERSORT, XCELL, EPIC, QUANTISEQ and TIDE. Interestingly, PFKFB4 was significantly negatively correlated with CD8 + T cells (include naïve, central memory, and effector memory), hematopoietic stem cells, and M2 macrophage (anti-inflammatory subsets of macrophage) in most cancer types (Fig. 5A), but it was positively related with neutrophils, cancer-associated fibroblasts (CAF), myeloid-derived suppressor cells (MDSCs), and M0 macrophage in various cancer types (Fig. 5B). These data strongly suggested that PFKFB4 induces a pro-inflammatory microenvironment via mediating cytokine release to recruit neutrophils, CAF, MDSCs and also suppresses CD8 + T cells and M2 macrophages in tumor microenvironment.

### Discussion

Currently, the primary challenge in the management of CRS is to identify more effective targets for specific therapeutic intervention while maintaining the therapeutic efficacy of CAR T-cells. Our study explored the genomic determinants which trigger cytokine release in immunotherapy and several other diseases. We found that PFKFB4 gene and glycolytic pathway activity were gradually upregulated with the development of increasing severity of cytokine release syndrome in CAR T-cell therapy. Moreover, these results were further validated in cohorts of people with COVID-19, influenza, autoimmune diseases and in tumor tissues although there was no change in glycolytic activity in flu infection and systemic lupus erythematosus (SLE).

Cytokines are regulators of the immune response to infection and inflammation. They function as a double-edged sword in that cytokines commonly alert immune cells to the presence of infections and tissue damage [37], however persistent cytokine production can, in turn, stimulate immune cells to secrete more cytokines that work in both autocrine and paracrine manners leading to a chronic inflammation state and even caused severe cytokine release syndrome when the immune system is hyperactivated. Therefore, it is important to find trigger factors to control cytokine release and maintain normal levels. To date, cytokine release has been increasingly explored in different fields, such as viral infection, autoimmune diseases, and immunotherapy. Most studies report that the JAK-STAT, NF-κB, and type I IFN signaling pathways are the main factors mediating the cytokine release process [38–40]. FDA-approved drugs targeting these pathways have already been used for the treatment in patients with cytokine release syndrome in CAR T-cell therapy, SARS-CoV-2 infection, and autoimmune diseases [39, 41–43]. In this study, we also found the activity of JAK-STAT signaling pathway was enhanced when patients developed higher grades of CRS, but not that of NF-κB and type I IFN signaling pathways. Furthermore, we found that the glycolytic activity was more noticeably upregulated than the other signaling pathways. Thus, drugs targeting glycolytic pathway may be beneficial for patients who have less/no response to anti-IL6 antibody, JAK inhibitors. Moreover, for the clinical management of cytokine release syndrome a combination of drugs targeting different pathways might be more effective than single-treatment approaches.

To date, most studies of PFKFB4 have focused on its increased expression in cancer tissues and its role in carcinogenesis [44–46], there is little knowledge about the biological mechanism on its upregulation under aforementioned situations. This gene encodes a bifunctional enzyme with kinase/phosphatase activity that is the most potent regulator of the PFK-1 gene, which is a key rate-limiting enzyme of glycolysis [47]. It has been reported that immune cells will adapt their metabolism upon infection and become highly glycolytic. For example, the SARS-CoV-2 infection triggers mitochondrial ROS production, which induces stabilization of hypoxia-inducible factor-1α (HIF-1α) and consequently promotes glycolysis [48]. As an enzyme involved in glycolysis, PFKFB4 gene may also change its expression level to deal with the situation. Our study is the first to report that the expression of PFKFB4 increases under these conditions and is a driving force in triggering cytokine release through the involvement of  glycolytic pathway activity in CAR T-cells, viral infections and autoimmune diseases. Interestingly, PFKFB4 may even regulate the infiltration of immune cells in tumor tissues, which suggests that PFKFB4 may also be a promising target for the regulation of tumor immunity in some types of cancer. Given the apparent role of PFKFB4 in cytokine release, further studies aimed at developing effective drugs target on PFKFB4 and glycolysis appear particularly promising.

Unlike transcription factors, which can regulate gene expression through direct binding to gene promoters, PFKFB4 is an enzyme with kinase and phosphatase activity. The key point is to find the transcription factor to bridge the PFKFB4 enzyme and cytokine gene expression. One study suggests that PFKFB4 ectopic expression elevates lactate levels (synthesized from pyruvate, which is the final product of glycolysis) in the culture medium which initiates NF-κB activation and nuclear translocation. NF-κB within the nucleus binds to the IL-6 promoter region and then enhances IL-6 expression [49]. Another study found  that PFKFB4 could interact with ICMT, a post-translational modifier of RAS, and activate RAS/AKT signaling pathway [50]. Several reports have implicated RAS in the ability to promote the production of inflammatory cytokines and chemokines (IL-6, IL-8, GM-CSF et al.) [51–54]. These results suggest that  investigations concerning how PFKFB4 engages in cytokine release in CAR T-cell, primary T cells, and assorted immune cells should be conducted in the future.

Our study has some limitations. First, we only evaluated 43 pre-infusion CD22 CAR T-cell products. Analyzing more samples will not only enhance the statistical power, but will also increase the possibility of finding more candidate driving genes. For this reason we used public datasets to validate our findings. However, the use of these public datasets brings along another limitation,since these public datasets and related background information included in them were out of our control. This limitation makes it difficult to explain some results from these datasets. For example, we saw that the PFKFB4 gene was upregulated in SLE patients with no change in glycolytic activity, but we could not provide a reasonable explanation for this finding since  we had no access to additional clinical information concerning these SLE patients. Another limitation is that we did not obtain information about the pro-inflammatory and anti-inflammatory cytokines levels in each patients’ serum after infusion of the CD22 CAR T-cell products or in the patients with the other diseases. This information would have helped to better understand which cytokines were mediated by PFKFB4 gene.

In summary, our results strongly indicate that PFKFB4 is a promising target for controlling cytokine release in immunotherapy and other cytokine release related diseases. More effort should be focused on the identification and development of drugs that target PFKFB4 and glycolysis.

## Supplementary Information

**Additional file 1: Figure S1.** Signaling pathway activity in different CRS grade groups. Associated signaling pathway score was calculated based on GSVA analysis. No obvious trend or statistical significance in these pathways. x-axis represent different CRS grade.

**Additional file 2: Figure S2.** Glycolytic activity in influenza infection and autoimmune disease. Glycolytic activity score in influenza infected diseases based on early and late stages (**A**–**C**). Glycolytic activity score in different stages of SLE (**D**) and SJIA (**E**).

**Additional file 3: Figure S3.** Association between PFKFB4 expression and survival time in cancer. Correlation between PFKFB4 expression and overall survival time in patients with liver hepatocellular carcinoma (**A**) and renal cancer (**B**). The x-axis the represents overall survival time. The y-axis represents survival probability. The different colors indicate the expression level of PFKFB4. (**C**). Expression correlations between PFKFB4 and genes involved in the glycolytic pathway.

**Additional file 4: Table S1.** Differentially expressed genes based on different CRS grade group.

## Data Availability

All 43 RCC files from Nanostring assay are available in GEO online dataset (GSE200296) until Dec 31, 2022.
