# Supplementary figures and images for: Identification of genomic determinants contributing to cytokine release in immunotherapies and human diseases
Source: J Transl Med. 2022 Jul 28;20:338. doi: 10.1186/s12967-022-03531-3 (PMC9331024; doi:10.1186/s12967-022-03531-3)

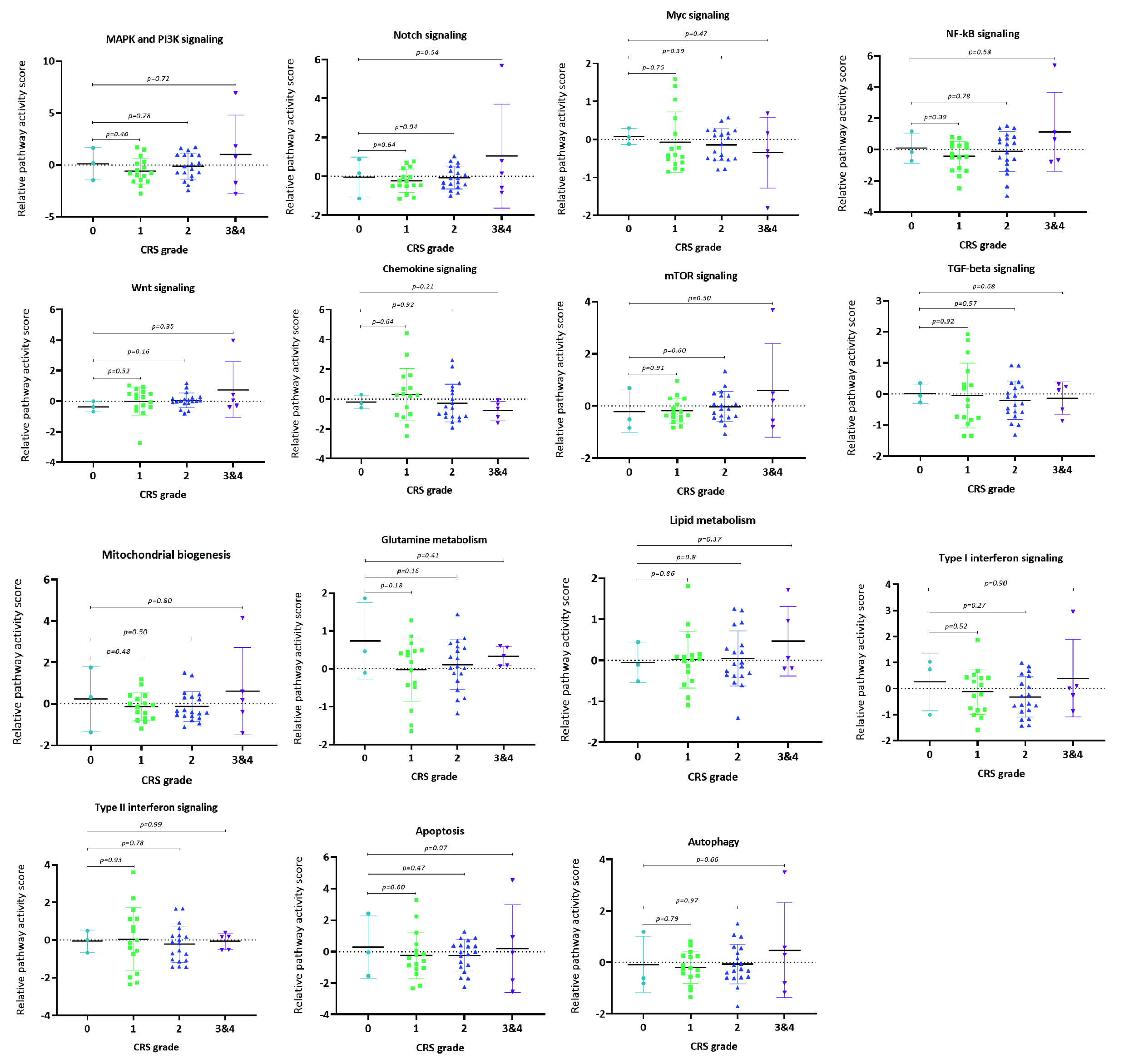

Supplement: Supplementary file 1 — Additional file 1: Figure S1. Signaling pathway activity in different CRS grade groups. Associated signaling pathway score was calculated based on GSVA analysis. No obvious trend or statistical significance in these pathways. x-axis represent different CRS grade. [file 12967_2022_3531_MOESM1_ESM.tif]

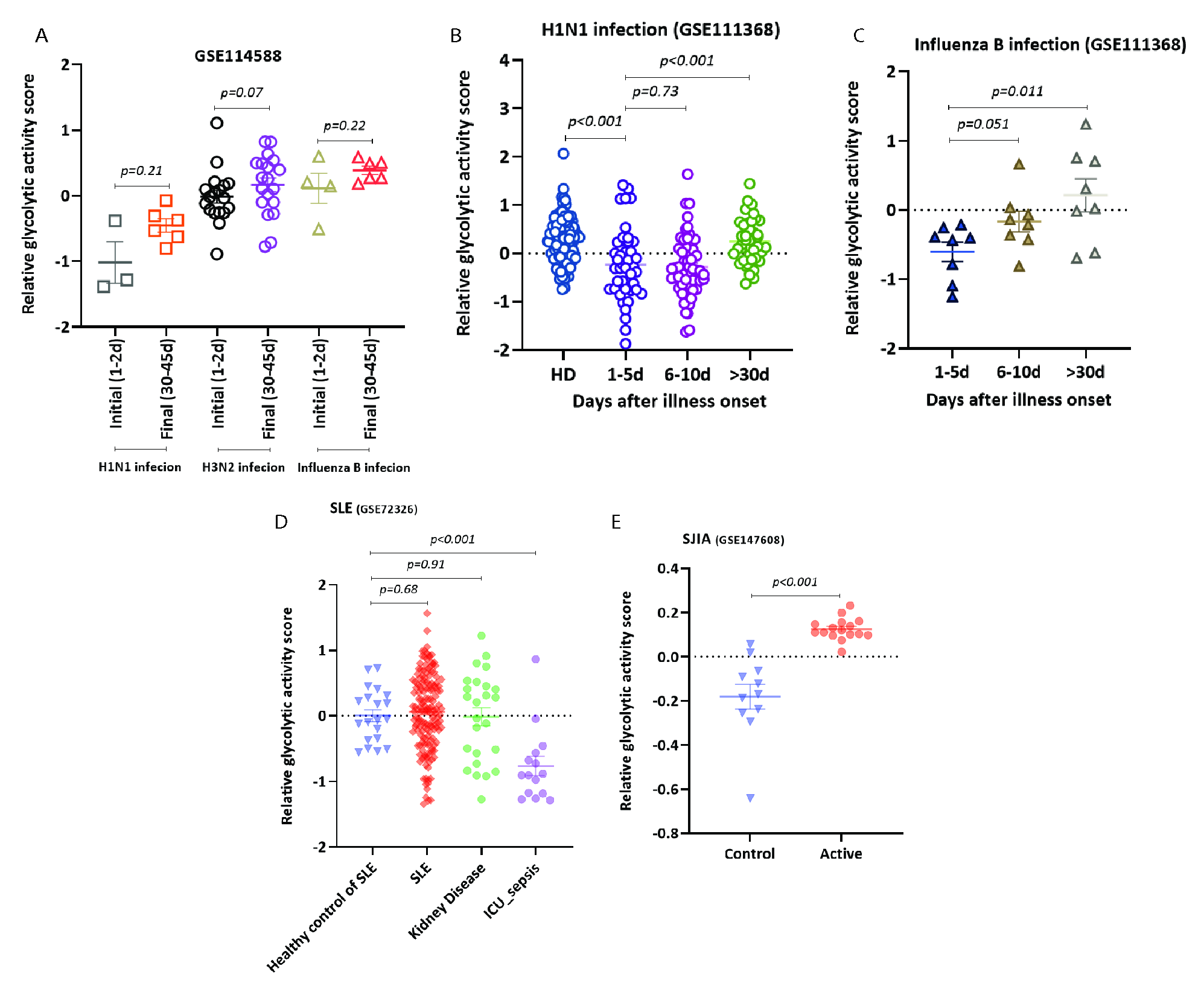

Supplement: Supplementary file 2 — Additional file 2: Figure S2. Glycolytic activity in influenza infection and autoimmune disease. Glycolytic activity score in influenza infected diseases based on early and late stages (A–C). Glycolytic activity score in different stages of SLE (D) and SJIA (E). [file 12967_2022_3531_MOESM2_ESM.tif]

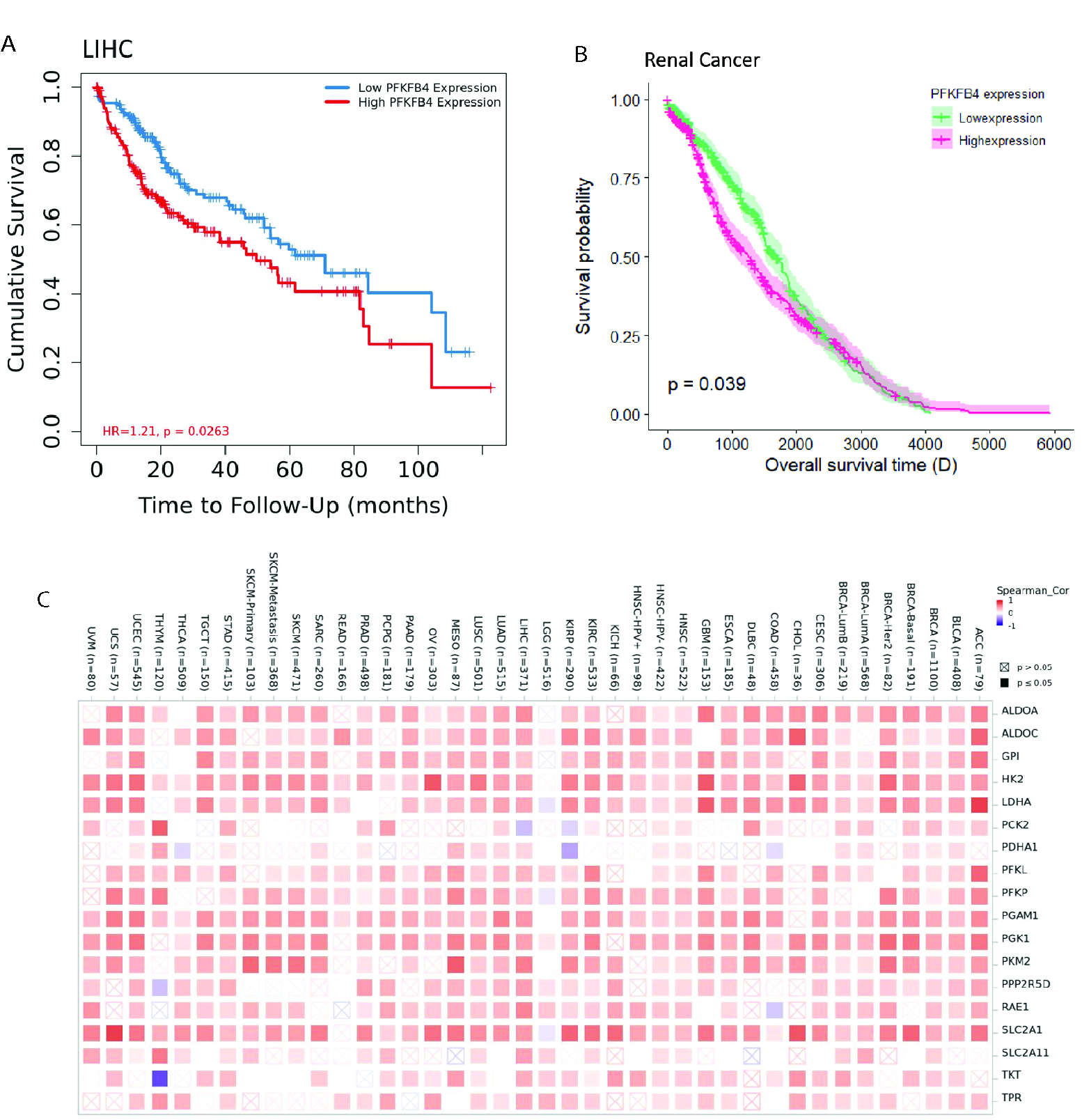

Supplement: Supplementary file 3 — Additional file 3: Figure S3. Association between PFKFB4 expression and survival time in cancer. Correlation between PFKFB4 expression and overall survival time in patients with liver hepatocellular carcinoma (A) and renal cancer (B). The x-axis the represents overall survival time. The y-axis represents survival probability. The different colors indicate the expression level of PFKFB4. (C). Expression correlations between PFKFB4 and genes involved in the glycolytic pathway. [file 12967_2022_3531_MOESM3_ESM.tif]
